# Putting Fungal Biology and Biotechnology to the test

**DOI:** 10.1186/s40694-023-00156-z

**Published:** 2023-04-18

**Authors:** Vera Meyer, Alexander Idnurm

**Affiliations:** 1 Chair of Applied and Molecular Microbiology, Institute of Biotechnology, Technische Universität Berlin, Straße des 17. Juni 135, 10623 Berlin, Germany; 2 School of BioSciences, The University of Melbourne, BioSciences 2, Parkville Campus, Parkville, VIC 3010 Australia

The San Francisco Declaration on Research Assessment (DORA) will celebrate its 10th anniversary in May 2023, a global initiative of researchers and institutions that “works with and in the community to raise awareness and support the development of best practices in research assessment” [1]. Worldwide, 2681 organisations and 19,987 individuals have signed the declaration so far, including our *almae matres* Technische Universität Berlin and the University of Melbourne [1]. In 2023 it will also be 10 years ago that we were both involved in founding the first open access journal in the research field of fungal bio(techno)logy, together with another signatory of DORA that being BioMed Central (now part of Springer Nature). It took a year until the inaugural issue of *Fungal Biology and Biotechnology* was published in 2014 [2]. What does DORA have to do with the BMC journal *Fungal Biology and Biotechnology*?

The signatories of DORA, which was drafted in 2012 during an annual meeting of the American Society for Cell Biology held in San Francisco, criticize journal-based metrics such as the Journal Impact Factor (JIF) to be used as primary parameter to evaluate the quality and impact of scientists, institutions, and scientific journals, and the use of these metrics for decisions on funding, hiring, promotion and tenure. According to DORA, “it is critical to understand that the Journal Impact Factor has a number of well-documented deficiencies as a tool for research assessment. These limitations include: (A) citation distributions within journals are highly skewed [3–5], (B) the properties of the Journal Impact Factor are field-specific: it is a composite of multiple, highly diverse article types, including primary research papers and reviews [3, 6], (C) Journal Impact Factors can be manipulated (or “gamed”) by editorial policy [7] and (D) data used to calculate the Journal Impact Factors are neither transparent nor openly available to the public [6, 8, 9]” [1]. In expressing these concerns, there was a focus on overvaluing the JIF when evaluating researchers or institutions that thus inhibits the development of Open Science, since researchers aim to publish in journals with a high JIF (i.e. journals with high citation numbers on average most of which are Closed Access) without taking into account whether the article is published as Open Access, how large or small a specific research community is or whether supplementary research data are published. Many journals have directly, or indirectly, adopted measures to address these criticisms.

Instead, DORA advocates evaluating the quality of the research itself, i.e. the scientific content of a publication is more important than the journal in which it is published, recommends online publications with no limits on the number of words, figures, and references in articles, and proposes that a variety of journal-based and article-based metrics shall be used to provide scientists a richer view of journal and article impact.

DORA, the worldwide Open Access movement, the unfortunate uncontrolled rise of ‘predatory journals’, a major gap in applied aspects of mycology and the associated expressions of woe by colleagues were the impetus for us to launch the first Open Access journal devoted to fungal bio(techno)logy. A decade later, we think that it is now the right time to evaluate the performance of *Fungal Biology and Biotechnology*, to discuss what has been achieved, highlight some of the most influential articles, and what further progress we envision for the years to come.

In our opening editorial we stated that “We intend for the journal to become a hub for researchers seeking information on their favorite topics as well as considering new or alternative directions. The journal shall become a platform for scientists from academia and industry to present their hottest findings in unicellular or multicellular fungal systems, in medical or industrial strains, and in so far unexplored species. This will be a platform for experts to discuss their visions on how fungi can help us to address some of the key challenges of the twenty-first century.” [2]. Before evaluating whether some of these goals have been achieved within the first decade, we will summarize the main statistics that are available on the journal performance.

*Fungal Biology and Biotechnology* publishes about 15–20 paper a year with a yearly acceptance rate that ranges between 40–60% of submitted manuscripts. The full text of all articles is deposited in digital archives around the world to guarantee long-term digital preservation. All articles are Open Access and made available under the Creative Commons Attribution (CC-BY) license, which means they are accessible online without any restrictions and can be re-used in any way if properly cited. The journal’s publication rate of 15–20 paper a year is too low to become considered by one of the, arbitrary, criteria of Clarivate for indexing with a JIF. However, all articles published in *Fungal Biology and Biotechnology* are included in PubMed, PubMed Central, the Directory of Open Access Journals and, since 2018, in Scopus^®^ that is a citation and abstract database provided by Elsevier and that records more than 22,000 currently peer-reviewed journals.

Scopus also creates several metrics that evaluate a journal’s citation impact by different standards to Clarivate’s JIF. These are described in the following sections and how *Fungal Biology and Biotechnology* has performed.

**Fig. 1:**
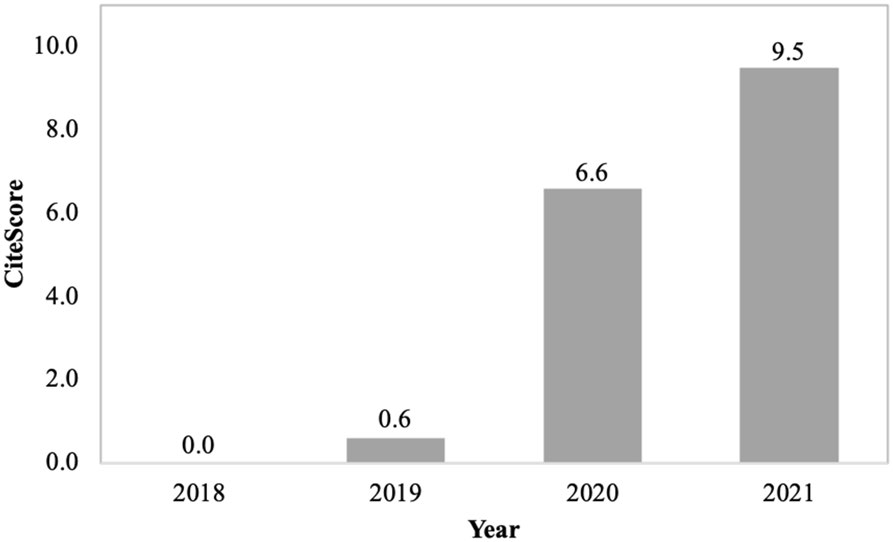
The CiteScore for *Fungal Biology and Biotechnology* has consistently risen since the journal was first indexed by Scopus

CiteScore is calculated by Elsevier, based on their Scopus database, and offers an alternative to Journal Impact Factors. For the numerator, the 2022 CiteScore counts the citations received in 2018–2021 to documents published in 2018–2021; the denominator is the number of documents published in these years. For *Fungal Biology and Biotechnology*, the CiteScore = 9.5, with an increase in trajectory since being included in Scopus (Fig. 1). Table 1 puts these scores in context by comparing them to those of several other mycology and microbiology titles.

**Table 1:** Comparison of different metrics between microbiology journals (in alphabetical order) with a focus on fungal biology

Journal title	CiteScore (2021)	SNIP (2021)	SJR (2021)
*Applied and Environmental Microbiology*	7.8	1.281	1.133
*Applied Microbiology and Biotechnology*	8.8	1.327	0.987
*Biotechnology for Biofuels and Bioproducts*	11.5	1.575	1.326
*Frontiers in Bioengineering and Biotechnology*	5.4	1.165	0.925
*Frontiers in Microbiology*	8.2	1.497	1.314
*Fungal Biology and Biotechnology*	9.5	1.844	1.185
*Fungal Genetics and Biology*	6.6	0.997	0.883
*Journal of Fungi*	4.1	1.6	0.98
*mBio*	11.2	1.742	2.767
*Microbial Cell Factories*	8.9	1.499	1.113
*Scientific Reports*	6.9	1.389	1.005

The 4-year CiteScore time window was chosen to fit all subject areas (Table 2). A 4-year publication window is long enough to capture the citation peak in the majority of disciplines. Based on these estimates, *Fungal Biology and Biotechnology* performs well above average in multiple disciplines, most notably “Ecology, Evolution, Behavior and Systematics” which is curious given the emphasis that could have been placed in biotechnology disciplines and likely a reflection of the wider appeal of the journal.

**Table 2:** Ranking of *Fungal Biology and Biotechnology* relative to other journals within specific categories

Category name	Rank	Percentile
Ecology, evolution, behavior and systematics	#31/687	95th
Applied microbiology and biotechnology	#13/118	89th
Biotechnology	#34/293	88th
Molecular biology	#80/386	79th
Cell biology	#58/274	78th

**Fig. 2:**
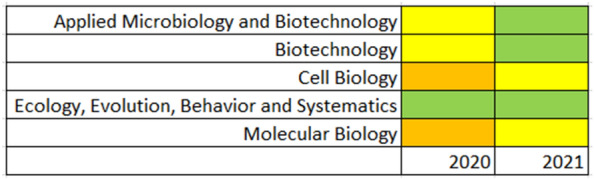
Scimago Journal Rank (SJR) for *Fungal Biology and Biotechnology*. The color coding indicates a quartile rank: legend: Top quartile: green —quartile 2: yellow—quartile 3: orange—bottom quartile: red (n/a for the journal)

The Scimago Journal Rank (SJR), which is based on Elsevier’s Scopus database, calculates the number of citations in one year to all journal’s articles in the preceding three years, weighted by the importance or prestige (calculated by a SJR algorithm) of the citing journals. Similar to the 4-year CiteScore, the SJR also places the journal in a number of top quartile disciplines, and with an upwards trajectory over the last two years (Fig. 2).

**Fig. 3:**
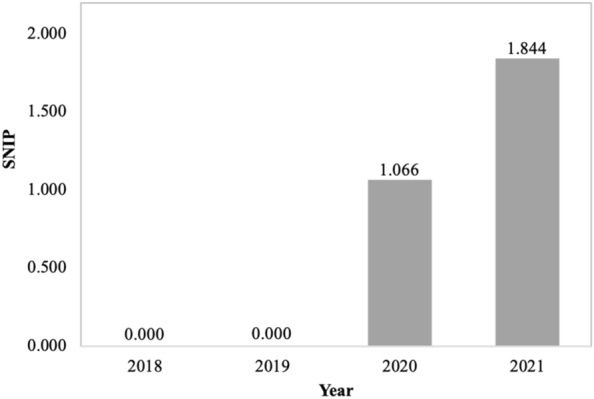
Source Normalized Impact per Paper (SNIP) illustrates the rising interest in manuscripts published in *Fungal Biology and Biotechnology*

The calculation of the Source Normalized Impact per Paper (SNIP), also Scopus-based, starts off similarly as for the SJR but then contextualizes and normalizes a journal’s citation-based impact by taking into account the total number of citations in a research discipline. Effectively, in a field where reference lists tend to be shorter, each citation counts more (and vice versa). A SNIP value of 1.0 represents the median (not the mean) number of citations for journals in a given field. Figure 3 illustrates, as with other metrics, an increase in this value.

However, the use of alternative metrics based on overall journal citations likely faces the same types of criticisms as raised for the use of the JIF. It may be more interesting to see what individual publications are most popular in *Fungal Biology and Biotechnology*. Table 3 includes details about the top 10 requests for papers up to late 2021. Some of these articles have received interest, with over 10,000 downloads each in a year, indicating a readership of the journal well beyond the scopes of its title. Even today, the journal’s first publication—a research article exploring fungal biodiversity for organic acid production in filamentous fungi—is amongst the most downloaded articles in the most recent year for the journal [10].

**Table 3:** The top 10 most accessed papers in *Fungal Biology and Biotechnology* in 2021

Article title	Authors	Year	Article requests (2021)
Growing a circular economy with fungal biotechnology: a white paper	Meyer et al	2020	12,008
Fungi as source for new bio-based materials: a patent review	Cerimi et al	2019	10,451
How a fungus shapes biotechnology: 100 years of *Aspergillus niger * research	Cairns et al	2018	5620
The beauty and the morbid: fungi as source for inspiration in contemporary art	Nai and Meyer	2016	3529
Invasive growth of *Aspergillus oryzae* in rice koji and increase in nuclear number	Yasui et al	2020	3164
Current challenges of research on filamentous fungi in relation to human welfare and a sustainable bio-economy: a white paper	Meyer et al	2016	2920
Interaction between arbuscular mycorrhizal fungi and *Bacillus* spp. in soil enhancing growth of crop plants	Nanjundappa et al	2019	2706
Vegan-mycoprotein concentrate from pea-processing industry byproduct using edible filamentous fungi	Souza-Filho et al	2018	2701
Potential of truffles in nutritional and medicinal applications: a review	Lee et al	2020	2694
Exploring fungal biodiversity: organic acid production by 66 strains for filamentous fungi	Liaud et al	2014	2403

The curious aspect about the popular papers in Table 3 are their topics. Nine of them are related to applications of fungi towards commercial outputs, in some cases specific fungal products, through to the top paper that is a broader coverage of using multiple fungi to improve sustainability. However, the 4th most read paper is about fungi used in art, which some might claim is just as important contribution by fungi as, e.g. citric acid production.

But it is not just the ‘top of a list’ that represents a journal, and *Fungal Biology and Biotechnology* has been proactive in providing, as in the DORA philosophy, a publishing home for research addressing the journal’s core focus. This divergence from a mainstream set of fungal topics carries over into different ways to communicate the research discoveries and the potential of fungi in society. In addition to regular articles, other ways the journal has been proactive in communicating findings published in the journal but also fungal biology across the discipline more widely has been through a blog, organized by Kustrim Cerimi and at times highlighting up and coming new scientists and their unpublished work presented at conferences, and its Twitter account (@FBBiotech), that has been organized by early career scientists Corrado Nai and Carsten Pohl.

The journal has also been active in the sponsorship of conferences to support its community base, including at the two big international meetings, being the Fungal Genetics Conference organized by the Genetics Society of America and held every two years at the Asilomar Conference Center in California and in the alternative year the European Fungal Genetics Conference that is held in different locations each time. The journal has been able to provide support for student prizes, and at the Asilomar Fungal Genetics Conference organized meetings between the editorial board members who were attending.

As editors, what can we draw from a decade of experiences? The numbers suggest that *Fungal Biology and Biotechnology* despite is small volume of output has had large impacts into the fungal publishing world and to mycology. Where into the next decade? Perhaps the style of articles reflects the personalities of the editors, whose terms in these roles are not indefinite and the journal may therefore change in direction. Broadening the ‘other’ contributions would be ideal, e.g. despite growing interest by the general public in fungi likely driven by a number of charismatic personalities promoting fungi globally, fewer and fewer institutions offer subjects devoted to this topic. A resource that could be filled would be in generating educational material that could be freely accessed and help restore this gap.
